# Acute thrombosis of the superior mesenteric artery in a 39-year-old woman with protein-S deficiency: a case report

**DOI:** 10.1186/1752-1947-5-17

**Published:** 2011-01-18

**Authors:** Nicola Romano, Valerio Prosperi, Giancarlo Basili, Luca Lorenzetti, Valerio Gentile, Remo Luceretti, Graziano Biondi, Orlando Goletti

**Affiliations:** 1 General Surgery Department, Health Unit Five, "F. Lotti" hospital Pontedera, Pisa, Italy

## Abstract

**Introduction:**

Acute thromboembolic occlusion of the superior mesenteric artery is a condition with an unfavorable prognosis. Treatment of this condition is focused on early diagnosis, surgical or intravascular restoration of blood flow to the ischemic intestine, surgical resection of the necrotic bowel and supportive intensive care. In this report, we describe a case of a 39-year-old woman who developed a small bowel infarct because of an acute thrombotic occlusion of the superior mesenteric artery, also involving the splenic artery.

**Case presentation:**

A 39-year-old Caucasian woman presented with acute abdominal pain and signs of intestinal occlusion. The patient was given an abdominal computed tomography scan and ultrasonography in association with Doppler ultrasonography, highlighting a thrombosis of the celiac trunk, of the superior mesenteric artery, and of the splenic artery. She immediately underwent an explorative laparotomy, and revascularization was performed by thromboendarterectomy with a Fogarty catheter. In the following postoperative days, she was given a scheduled second and third look, evidencing necrotic jejunal and ileal handles. During all the surgical procedures, we performed intraoperative Doppler ultrasound of the superior mesenteric artery and celiac trunk to control the arterial flow without evidence of a new thrombosis.

**Conclusion:**

Acute mesenteric ischemia is a rare abdominal emergency that is characterized by a high mortality rate. Generally, acute mesenteric ischemia is due to an impaired blood supply to the intestine caused by thromboembolic phenomena. These phenomena may be associated with a variety of congenital prothrombotic disorders. A prompt diagnosis is a prerequisite for successful treatment. The treatment of choice remains laparotomy and thromboendarterectomy, although some prefer an endovascular approach. A second-look laparotomy could be required to evaluate viable intestinal handles. Some authors support a laparoscopic second-look. The possibility of evaluating the arteriotomy, during a repeated laparotomy with a Doppler ultrasound, is crucial to show a new thrombosis. Although the prognosis of acute mesenteric ischemia due to an acute arterial mesenteric thrombosis remains poor, a prompt diagnosis, aggressive surgical treatment and supportive intensive care unit could improve the outcome for patients with this condition.

## Introduction

Acute thromboembolic occlusion of the superior mesenteric artery (SMA) is a condition with a serious prognosis [1]. Acute mesenteric ischemia (AMI) is an uncommon occurrence and represents 0.1% of hospital admissions [2]. Despite considerable advances in medical diagnosis and treatments over the past four decades, mesenteric vascular occlusion still has a poor prognosis, with an in-hospital mortality rate of 59 to 93% [3]. The high rate of mortality can be explained by the nonspecific signs and symptoms that characterize AMI. The classic teaching of "pain out of proportion to physical examination findings" is often seen during the early stage of ischemia when the abdomen is soft and not tender. Distention and severe tenderness with rebound guarding appear as a consequence of the bowel infarction [2]. The serologic markers cannot aid in the diagnostic process because they are nonspecific (inorganic phosphate, lactic acid, aldolase, creatinine kinase, and alkaline phosphate) [2]. An elevated white blood cell (WBC) count (leukocytes measuring over 15,000 cells) is a common, but unspecific, finding [2]. According to Kurland [4], another nonspecific sign is metabolic acidosis. Treatment of this condition is focused on early diagnosis, surgical or intravascular restoration of blood flow to the ischemic intestine, surgical resection of the necrotic bowel, and supportive intensive care.

One aspect that influences survival is the cause of the bowel ischemia, which can be classified as a non-thrombotic or a thrombotic event [5]. Conditions that cause nonthrombotic mesenteric ischemia (NOMI) include a low-flow state (for example, cardiogenic shock, pancreatitis, sepsis, hypovolemia), mechanical causes (for example, strangulated hernia, adhesive bands, intussusceptions), and colon ischemia after aortic aneurysm repair [5]. NOMI represents 25% of the causes of the AMI [2]. The specific thrombotic conditions include arterial embolization (superior mesenteric artery embolization; SMAE), arterial thrombosis (superior mesenteric artery thrombosis; SMAT), and mesenteric venous thrombosis (acute mesenteric venous thrombosis; AMVT) [5]. The most common cause of AMI is SMAE, which represents 50% of the causes of AMI [2]. SMAT can be seen in 10% of the patients after AMVT [2]. These thromboembolic phenomena may be associated with prothrombotic disorders, such as protein C, protein S, and antithrombin III (AT III) deficiency [6]. In this report, we describe the case of a woman with a thrombophilic state, in whom a small bowel infarct developed because of an acute thrombotic occlusion of the SMA, involving the splenic artery as well.

## Case presentation

**Figure 1:**
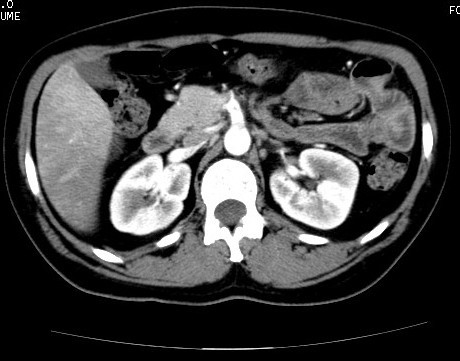
**Abdominal computed tomography scans**.

**Figure 2:**
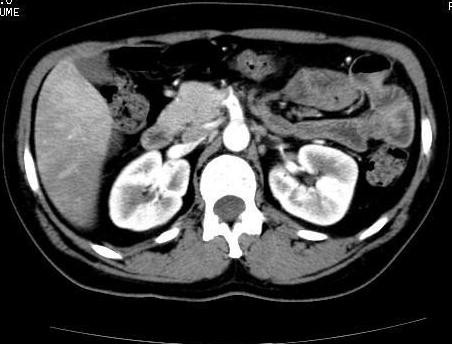
**Abdominal computed tomography scans**.

**Figure 3:**
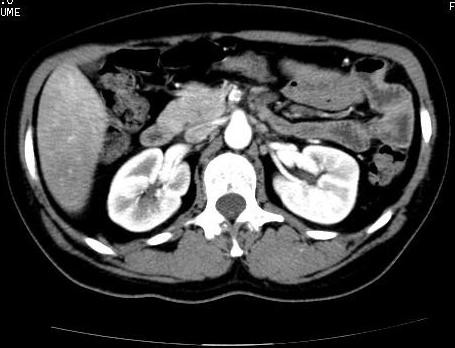
**Abdominal computed tomography scans**.

A 39-year-old Caucasian woman presented in our emergency department with acute abdominal pain associated with nausea, vomiting, and signs of intestinal occlusion. The clinical history of the patient highlighted two other admissions for the same clinical signs. During the first admission, she was given an abdominal computed tomography (CT) scan that demonstrated only the presence of free fluid localized in the pouch of Douglas and the perihepatic region. In relation to these signs, she was given an emergency, explorative laparotomy, with lavage of the abdomen. The laparotomy demonstrated only hyperemic jejunal and ileal handles. She was discharged after nine days without any complications. Two weeks after the patient was readmitted to the same hospital with similar symptoms, and she was treated with corticosteroids, mesalazine, and metronidazole with a complete resolution of the symptoms. Five days later, the patient was admitted to our unit. At admission, she had leukocytosis (WBC, 19.960 × 10^6^/L) and normal levels of the coagulation parameters. She was given abdominal ultrasonography in association with Doppler ultrasonography (Esaote Megas GPX 7.5-MHz convex probe), highlighting a thrombosis of the SMA. As a result of this clinical picture, she underwent an abdominal CT scan (Figures 1 to 3), demonstrating the presence of a partial thrombosis of the celiac trunk, a thrombosis of the SMA for a 25- to 30-mm tract, and the lack of a splenic artery. She immediately underwent an explorative laparotomy, showing ischemic, but viable handles, and a tree revascularization by thromboendarterectomy with a Fogarty catheter was performed. In the following postoperative days, she was given a scheduled second and third look, showing necrotic handles (the first jejunal handle, the last ileal handle, and about 20 cm of the medium ileum) in the first procedure, and another necrotic tract of small bowel (the other 10 cm of the first jejunal tract) in the last procedure. During that surgical procedure, we performed duodenojejunal and three other laterolateral anastomoses to reestablish the bowel continuity. A T-tube was inserted to protect the duodenojejunal anastomosis. A cholecystectomy and biliary diversion were performed to reduce the biliary output. In relation to the risk of dehiscence, we performed a colonostomy in the right flank. During all the surgical procedures, we performed intraoperatory Doppler ultrasound of the SMA and celiac trunk to control the arterial flow without evidence of a new thrombosis. The patient stayed in the ICU for 27 days with total parenteral nutrition and antibiotics therapy. A coagulation screening demonstrated a thrombophilic state for a protein-S (16%) deficiency with normal values of VIII, IX, and XI factors. The search for antiphospholipid antibodies was negative, and the genetics test for factors II to V and methylenetetrahydrofolate reductase (MTHFR; the deficiency of this enzyme is associated with an increased risk to develop massive thromboembolic events) was negative (no mutations). She was discharged from our unit after 37 days without any complications. After three months, the patient had a surgical procedure for restoring the bowel continuity. The patient was evaluated after one week, and one, three, and six months after discharge with blood and coagulation examinations, abdominal ultrasonography, Doppler ultrasound, and abdominal CT scan. She was asymptomatic and stayed well. At one year, we had successfully restored the bowel continuity without complications.

## Discussion

Acute mesenteric ischemia is a rare abdominal emergency that usually requires wide intestinal resection and carries a high mortality rate (Table 1[7-13]) with the adverse effects of short-bowel syndrome in the surviving patients [6]. A critical point that influences the survival rate is prompt diagnosis in patients with AMI. Numerous surgical reports indicated that acute intestinal ischemia (AII) is associated with a poor prognosis [13]. The poor signs, symptoms, and nonspecific laboratory tests are among the causes of the delay in the diagnosis. Other examinations that can be helpful in the diagnostic process are angiography, computed tomography angiography (CTA), and magnetic resonance angiography (MRA) [2]. When no clinical evidence is found for an immediate surgical intervention, such as peritonitis or gastrointestinal hemorrhage, angiography could be considered the treatment of choice in patients with suspected AMI, because this investigation allows us to distinguish between nonthrombotic and thrombotic causes [14]. Moreover, angiography allows us to treat the occlusion with a restoration of the blood flow by using an endovascular approach, such as percutaneous transluminal angioplasty and thrombolysis [5-14].

**Table 1:** Comparative death rates for thrombotic causes of acute intestinal ischemia

	Arterial embolism			Arterial thrombosis		Venous thrombosis		Overall deaths	
Authors	Year	**No**.	%	**No**.	%	**No**.	%	**No**.	%
Ottinger [7]	1967	22/29	76	21/22	95	8/10	80	51/61	83
Smith [8]	1976	6/7	86	9/10	90	3/3	100	18/20	90
Kairaluoma [9]	1977	10/11	91	19/21	90	-	-	29/32	91
Hertzer [10]	1978	4/7	57	2/2	100	-	-	6/9	67
Sachs [11]	1982	9/14	64	12/12	100	4/11	36	25/37	68
Bergan [12]	1987	5/6	83	6/8	75	-	-	11/14	79
Klempnauer [13]	1997	16/21	76	22/27	81	11/30	37	49/78	62
Endan [5]	2000	13/22	59	13/21	62	2/15	13	28/58	48
Collated experience		85/117	74	104/123	86	28/69	53	217/309	73

Simo *et al*. [14] reported a 90% success rate for lysis of the embolus in patients with SMAE. However, although the endovascular approach may rapidly restore the blood flow to the bowel, the time needed for thrombolysis is variable, and the bowel viability cannot be assessed with laparotomy [14]. This can result in a diagnostic delay that can compromise other viable bowel tracts [5]. According to Kirkpatric [1], the CTA has shown a diagnostic sensitivity of 96% and a specificity of 94%. The magnetic resonance angiography (MRA) is another newer imaging technique that seems to be promising for the diagnosis of AMI, although this technique cannot help us to diagnose NOMI secondary to a low-flow state or to identify distal embolic disease [2]. Generally, the IMA is due to an impaired blood supply to the intestine caused by thromboembolic phenomena. These phenomena may be associated with a variety of congenital prothrombotic disorders (PDs), such as protein-C and protein-S deficiencies, AT III deficiencies (anti-phospholipid antibodies), Factor V Leiden (FVL), Prothrombin G20210A mutation, and C677T homozygous mutation of the *MTHFR *gene. The prevalence of these mutations differs among geographic areas and ethnic groups [6]. In our patient, we found deficiencies of the S protein, although some studies demonstrated a prevalence of this disorder in a Chinese population (59%) compared to a Caucasian population (15%)[6]. The level of S protein is higher in men than in women, but increases with age in women but not in men [16]. In women, the levels of an S protein are lower before menopause, while taking oral contraceptives, or while undergoing hormone-replacement therapy, and during pregnancies [16].

The International Society of Thrombosis and Haemostasis Standardization Subcommittee defined three n-types of hereditary S-protein deficiencies [16]. Type I is defined by low levels of free and total antigen with decreased APC cofactor activity [16]. Type II protein-S deficiency is characterized by normal levels of a free and total antigen, with low levels of APC cofactor activity [16]. Type III protein-S deficiency is defined by normal to low levels of total antigen, low free protein S, and an elevated fraction of protein S bound to C4BP [16]. The role of the protein S is based on an increase of the anticoagulant action of protein C [16]. Protein C is a proteinase that inactivates the coagulation factors V, Leiden, and VIII, and protein S increases the action of protein C [17]. The SMA normally serves as the primary arterial supply of the jejunum, the ileum, and the colon to the level of the splenic flexure [7].

Ottinger *et al*. [7] demonstrated a general correspondence between the site of the occlusion, the extent of the infarcted areas, and the prognosis [7]. To explain this concept, we can divide the SMA into four regions [7]. The first portion is the artery origin, and the second tract is represented by the main trunk, including the middle colic artery (MCA). Region three corresponds to the main trunk beyond the origin of the MCA, and the last region (IV) is the most peripheral portion of the SMA and its branches [7]. The occlusion of the SMA in the first region produces a more-extensive infarction than that when the site of occlusion is distal to the origin of some of its branches [7].

Another factor that influences the prognosis is the etiologic subsets [3]. We can grossly distinguish two different origins, thrombotic and non-thrombotic. Non-occlusive mesenteric ischemia, the more frequent non-thrombotic cause, is caused by low-flow states. The thrombotic condition includes arterial embolism, arterial thrombosis, and mesenteric venous thrombosis. According to Schoots [3], acute mesenteric ischemia due to a venous thrombosis has a better prognosis compared with arterial causes of MIA. In this case, the improved survival rate can be explained by the segmental bowel infarction and the need for limited intestinal resection. The poor prognosis of patients with mesenteric arterial occlusions is most likely due to the proximal location of the occlusion in the vessel tree; this determines a more extensive bowel infarction and the need for extended intestinal resection. A mesenteric arterial embolism results in a different extension of the infarcted areas because the emboli can occlude the vessel tree to different levels. The prerequisite for success of a revascularization is prompt diagnosis. The delay from the first examination to laparotomy was significantly shorter among the patients in whom the diagnosis was suspected; however, early diagnosis did not improve survival [1]. Moreover, Giulini [18] demonstrated a correlation between of prompt diagnosis of an AMI and survival. However, for the non-specific symptoms, during the early phase, the diagnosis is often delayed [19].

The second-look laparotomy remains the gold standard for the assessment of further bowel viability, and, at the same time, it is the only way to remove infarcted tracts of the bowel [20]. During the surgical procedure, the bowel viability can be assessed by the physical examination (inspection of bowel and palpation of the vessel) or by ultrasound examination and intravenous fluorescein [20]. Although the second-look laparotomy is the gold standard for the treatment of AMI, some authors perform a second-look laparoscopy to decrease the severe anesthesiologic and surgical trauma in these critically ill patients [20]. Levy *et al*. [20], in a series of 92 patients, underlined the beneficial role of the second-look laparoscopy in patients' survival.

## Conclusion

Acute thrombosis of the SMA represents a rare emergency in young female patients. Although in these patients, mesenteric infarction has a low incidence, acute thrombosis should be always suspected, especially in young female patients receiving therapy with estro-progestinic hormones and who show signs of an acute abdomen. These cases should be investigated with CT-angiography or, if feasible, with arteriography to exclude an acute mesenteric infarction. If the CT-angiography or the arteriography confirms this diagnosis, an early laparotomy should be performed.

In our case, we performed a second-look laparotomy because this surgical procedure allowed us to conduct a physical examination of the bowel and artery (for example, palpation of the vessels, inspection of the bowel, and evaluation of the anastomosis). Moreover, the second-look and other laparotomies suggest the performance of an intraoperatory Doppler ultrasound to evaluate the artery flow. According to Ottinger [7], a new thrombosis of the SMA can develop in the site of the arteriotomy during the first 48 hours. The possibility of evaluating the arteriotomy, during a repeated laparotomy with a Doppler ultrasound, is crucial; an early planned repeated laparotomy improves the prognosis of the surgical approach. Although the prognosis of the AMI due to an acute arterial mesenteric thrombosis remains poor, a prompt diagnosis, aggressive surgical treatment, and a supportive intensive care unit for a patient with AMI could improve the prognosis.

## Consent

Written informed consent was obtained from the patient for publication of this case report and accompanying images. A copy of the written consent is available for review by the Editor-in-Chief of this journal.

## Competing interests

The authors declare that they have no competing interests.

## Authors' contributions

NR wrote the article. VP researched and retrieved the bibliography. GB was the language supervisor. LL analyzed and interpreted the abdominal ultrasound data. VG acquired and interpreted the Doppler ultrasound data. RL contributed to writing the manuscript, controlling and correcting the general surgery portion. GB interpreted the hematology. OG supervised and was the chief of the team. All authors read and approved the final version of the manuscript.
